# The use of self‐report questionnaires in an analysis of the multidimensional aspects of pain and a correlation with the psychological profile and quality of life in patients with burning mouth syndrome: A case‐control study

**DOI:** 10.1111/joor.13343

**Published:** 2022-06-21

**Authors:** Federica Canfora, Elena Calabria, Giuseppe Pecoraro, Luca D′Aniello, Massimo Aria, Gaetano Marenzi, Pasquale Sammartino, Michele Davide Mignogna, Daniela Adamo

**Affiliations:** ^1^ Department of Neuroscience, Reproductive Sciences and Dentistry University of Naples Federico II Naples Italy; ^2^ Department of Social Sciences University Federico II of Naples Naples Italy; ^3^ Department of Economics and Statistics University Federico II of Naples Naples Italy; ^4^ Head and Neck Clinical Department AOU San Giovanni di Dio e Ruggi di Aragona Salerno Italy

**Keywords:** brief inventory, burning mouth syndrome, pain, pain, short‐form McGill pain questionnaire, visual analogue scale

## Abstract

**Background:**

The symptomatology in Burning Mouth Syndrome (BMS) is complex and it should be considered in accordance with a biopsychosocial model.

**Objectives:**

To evaluate the multidimensional aspects of pain with a complete battery of tests and to analyse its relationship with potential predictors such as mood disorders, sleep and quality of life.

**Methods:**

Forty patients with BMS versus an equal number of age and sex‐matched healthy controls were enrolled. The VAS, SF‐MPQ, BPI, PD‐Q, BDI‐II, STAI, PSQI, ESS, SF‐36 and OHIP‐14 were administered.

**Results:**

The scores of the VAS, SF‐MPQ, BPI, PD‐Q, BDI‐II, STAI, PSQI, SF‐36 and OHIP‐14 were statistically significantly higher in the BMS patients than the controls (*p* < .001**). A strongly linear correlation between pain (VAS, SF‐MPQ, BPI and PD‐Q) and disease onset (STAI, BDI‐II, PSQI and sub‐items of SF‐36 and OHIP‐14) was found. In the multiple regression analysis, the contributions of the BDI‐II and OHIP‐14 were found to be statistically significant with the SF‐MPQ, PD‐Q and BPI in terms of severity and interference, while the contributions of the STAI and sleep were found to be statistically significant with the SF‐MPQ and BPI in terms of severity and interference, respectively.

**Conclusions:**

Pain tests are differently correlated with mood and quality of life. Therefore, a complete analysis of the patient requires several tools to better understand the multidimensional aspects of pain in BMS.

## Graphical abstract

The symptomatology in Burning Mouth Syndrome (BMS) is complex and it should be evaluated considering the multidimensional aspects of pain in accordance with a biopsychosocial model. 40 patients with BMS versus 40 healthy controls were enrolled and the VAS, SF‐MPQ, BPI, PD‐Q, BDI‐II, STAI, PSQI, ESS, SF‐36 and OHIP‐14 were administered. The results showed that pain tests are differently correlated with mood and quality of life showing that a complete analysis of the patient requires several tools to better understand the multidimensional aspects of pain in BMS.

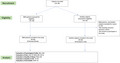

## 1 INTRODUCTION

Burning Mouth Syndrome (BMS) is a complex chronic oro‐facial pain disorder characterised by pain in the oral cavity without any evident clinically causative lesions. In accordance with the International Classification of Oro‐facial Pain, 1st edition (ICOP 2020),^1^ BMS is defined as an intra‐oral burning or dysaesthetic sensation, recurring daily for more than 2 h per day for more than 3 months, without evident causative lesions on clinical examination and investigation; including patients report not only bilateral intra‐oral or facial pain but also those with a unilateral symptomatology. The aetiology is unknown and is probably of multifactorial origin, with increasing evidence that BMS may be a neuropathic disorder involving the central and peripheral nervous system.^2^ The worldwide prevalence of the disease is around 4% but varies considerably in relation to the different definitions of BMS with consequent different inclusion criteria considered. The prevalence increases in post‐menopausal women (18%), with a female‐to‐male ratio ranging from 3:1 to 20:1.^3^ This is probably due to a reduction in the sexual hormones, which can cause a toxic effect on the peripheral and central neurons.

The main symptom reported by patients affected by BMS is pain.^4^ This is, therefore, the primary element to consider in any BMS diagnosis. The pain is usually persistent, of moderate intensity and poorly localised and described as dull, pressing or of a burning character, either scalding, tingling or numbing. It does not disturb sleep and is less severe in the morning, getting worse during the day, although generally alleviated by eating and drinking. The most common site for the pain or burning is the tongue (the anterior two‐thirds or the tip), followed by the hard palate, gingivae, lower lips and pharynx. The symptomatology is frequently complicated by additional symptoms such as dysgeusia, subjective xerostomia, a bitter/metallic taste, sialorrhea, globus and foreign body sensation.^3^

Pain manifested in the physical and anatomical dimensions is characterised by psychological, social and cultural dimensions that affect the subjective experience of pain in a biopsychosocial context. The complete analysis of the symptoms of BMS and of the way in which they can affect the life of the patient is very complex and continues to be a challenge for clinicians. For this reason, various questionnaires have been used in the assessment of pain in patients with BMS in order to evaluate every feature of this multidimensional symptomatology.^3^

Traditionally, the Visual Analogue Scale (VAS),^5^, ^6^ the Numeric Rating Scale (NRS)^7^ and the short form of the McGill Pain Questionnaire (SF‐MPQ)^8^, ^9^, ^10^ have been the most frequently used tools for measuring and estimating pain intensity and quality in BMS. In addition, recently, in three studies^11^, ^12^, ^13^ the Pain DETECT Questionnaire (PD‐Q) has been used in a screening stage to evaluate the neuropathic component of pain in this disease. However, none of these questionnaires are able to assess how the pain interferes with the patient's functioning. In contrast, the Brief Pain Inventory (BPI)^14^ is a simple and valid tool able to measure not only the intensity and location of the pain but also the degree of interference in the lives of patients with either nociceptive or neuropathic pain. Accordingly, it has been used only in our previous study and in the study of Lee et al.^15^

Until now, few studies have investigated the pain experience of patients affected by BMS through a comprehensive pain assessment including several questionnaires, taking into account also the psychological profile of the patients and analysing the potential predictors of pain.^16^, ^17^, ^18^ Therefore, the primary outcome of this study has been to investigate the intensity, quality and interference of the pain through the use of different questionnaires such as the VAS, SF‐MPQ, PD‐Q and BPI in order to obtain a comprehensive multidimensional analysis. The secondary outcome has been to evaluate the relation between pain, mood disorders (anxiety, depression and sleep disorders) and quality of life (QoL) to better understand, which are the most important predictors that may affect the experience of pain.

## 2 METHODS

### 2.1 Study design and participants

This was an observational case‐control study, which was conducted at the Oral Medicine Department of the University of Naples ‘Federico II’ in accordance with the ethical principles of the World Medical Association Declaration of Helsinki. It was approved by the Ethical Committee of the University (Approval Number: 251/19—the date of approval was February 20, 2019). The adopted methods conformed with the Strengthening of the Reporting of Observational Studies in Epidemiology (STROBE) guidelines for observational studies.^19^ It was conducted between March 2020 and December 2021.

The target sample size equal to 40 patients for each group (BMSs and controls) was set by fixing a power test value (1‐Beta) no less than 99% associated with a significance of no more than 1%. This sample size was carried out using the effect size value of 1.49, measured in a previously published research study regarding the PSQI scale.^20^ The calculations were computed using Gpower software (v 3.1.9).

At the beginning 90 participants were recruited, aged 55–75 years. They included patients from our group suffering from BMS at the first consultation who had never been treated and, in addition, healthy subjects presenting for routine dental treatment during the study period. Every eligible subject was invited to participate in this study and provided written informed consent. No payment was provided for participation. The patients with BMS and the controls were enrolled in order to match the sample by age, gender and educational level. At the baseline appointment (time 0), 46 patients in the study group and 44 in the control group were considered eligible for this study. At the end of this process, considering the target sample size, 40 individuals in each group met the inclusion and exclusion criteria.

In accordance with the International Classification of Oro‐facial Pain (ICOP 2020) 1st edition,^1^ the inclusion criteria of the BMS group were:

- patients experiencing an intra‐oral burning or dysaesthetic sensation, recurring daily for more than 2 h per day for more than 3 months, without evident causative lesions on clinical examination and investigation; the pain has the characteristics of burning quality and is experienced superficially in the oral mucosa;
- patients, male or female, aged at least 18;
- patients with normal blood test findings (including blood count, blood glucose levels, glycated haemoglobin, serum iron, ferritin and transferrin);
- patients who are not currently in treatment with psychotropic drugs.

The BMS group exclusion criteria were:

- patients suffering from diseases that could be recognised as a causative factor of BMS;
- patients unable to understand or complete the questionnaires;
- patients having a history of a psychiatric disorder or a neurological or organic brain disorder;
- patients undergoing treatment with psychotropic drugs or systemic drugs possibly associated with oral symptoms;
- patients having a history of alcohol or substance abuse;
- patients suffering from obstructive sleep apnoea syndrome (OSAS).

The inclusion criteria of the healthy subjects were:

- subjects without any lesion of the oral mucosa;
- subjects referring to the dental clinic for routine dental care without acute/chronic pain;
- subjects, male or female, aged at least 18;
- subjects without a psychiatric disorder or a neurological or organic brain disorder;
- subjects without a history of BMS;
- subjects with normal blood test findings (including blood count, blood glucose levels, glycated haemoglobin, serum iron, ferritin and transferrin);
- subjects who had not undergone treatment with psychotropic drugs.

The exclusion criteria of the healthy subjects were:

- subjects unable to understand or complete the questionnaires;
- subjects having a history of alcohol or substance abuse;
- subjects suffering from OSAS.

### 2.2 Measures

At their first consultation, all the participants underwent a careful medical analysis, specifically an intra‐ and extra‐oral examination by an expert clinician in oral medicine (DA). Their gender, age, years of education, family situation, job status, disease onset (in years), sleep duration (in hours), risk factors (current smoking status, alcohol consumption), oral symptoms and their location, medical comorbidities and systemic drugs taken were recorded and their Body Mass Index (BMI) was calculated.

**FIGURE 1:**
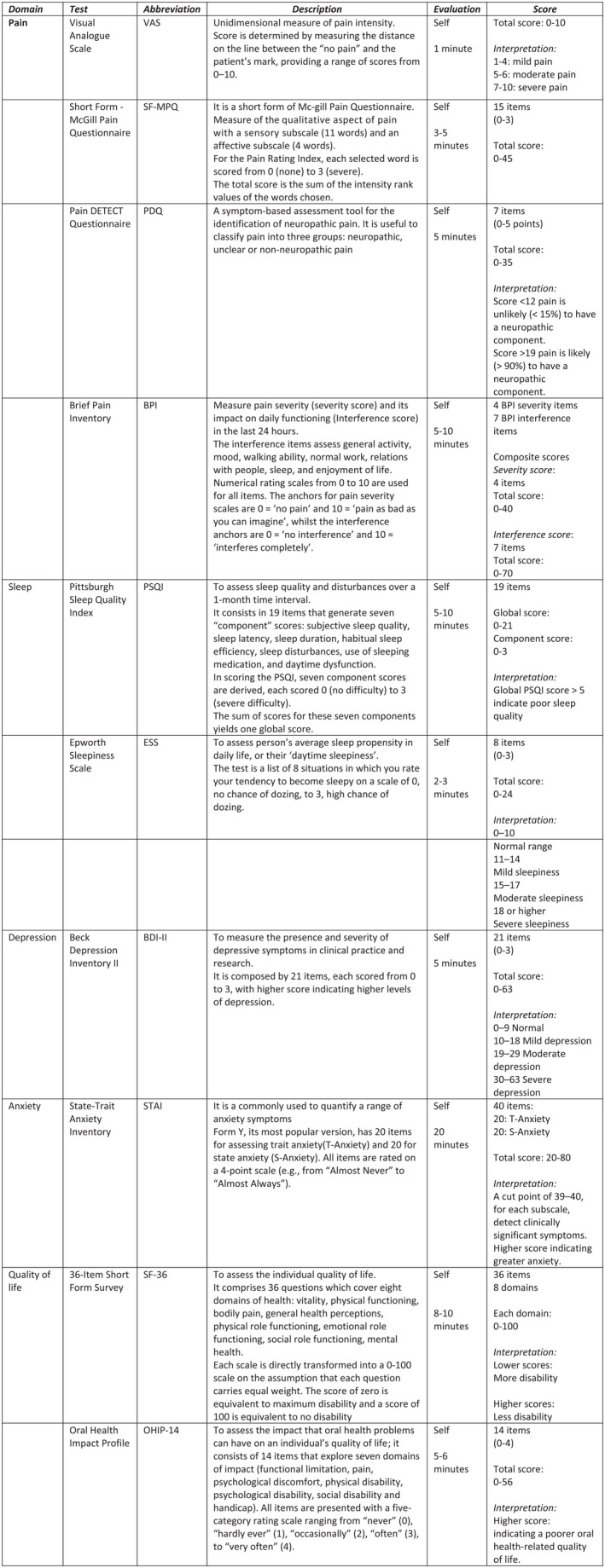
Psychometric tests battery. Tests explanation divided for domains: test, abbreviation, description, administration typologies and time needed, scoring interpretation

In addition, the patients and controls underwent the following battery scales for a complete analysis of any pain experienced and their psychological profile and QoL (Figure 1).

### 2.3 Pain assessment

The Visual Analogue Scale (VAS)^21^ is a validated instrument used to measure the intensity of pain. Patients mark a 10 cm scale ranging from the absence of pain (at the 0 cm end) to maximum pain (at the 10 cm end); the distance between 0 and the patient's mark indicates the intensity of the pain suffered.

The short form of the McGill Pain Questionnaire (SF‐MPQ)^22^ is a measure of the quality of pain. It is a multidimensional pain questionnaire, which measures the sensory, affective and evaluative aspects of the perceived pain.^23^ It comprises 15 items from the original MPQ, each scored from 0 (none) to 3 (severe). There are no established critical cut‐off points for the interpretation of the scores and, as for the MPQ, a higher score indicates worse pain.

The Pain DETECT Questionnaire (PD‐Q)^11^ is a reliable screening tool with a high sensitivity and specificity for the identification of neuropathic pain.^24^ It is a self‐reported questionnaire useful to classify pain into three groups: neuropathic, unclear or non‐neuropathic pain. It consists of nine items: seven descriptive items describe the patient's sensorial experience rated on a scale from 0 to 5 (never, hardly, slightly, moderately, strongly and very strongly noticed), one item for the pain radiating pattern (0 to 2 points), and one item for the pain temporal pattern (−1 to 1 point). For diagnostic purposes, a validated algorithm was used to calculate a total score ranging from 0 to 38. A score of less than 12 indicates the presence of nociceptive pain, a score of 12–19 is suggestive of possible neuropathic pain while a score of over 19 confirms certain neuropathic pain.^11^

The Brief Pain Inventory (BPI)^14^ is a validated and widely used inventory that has been developed to assess the severity of pain and the interference of pain.^25^ It is a 9‐item self‐administered questionnaire in which the pain severity is assessed by 4 items, including the worst and least severe pain experienced in the previous 24 h, the pain severity on average and the pain ‘right now’, ranging from 0 (no pain) to 10 (pain as bad as you can imagine). The pain‐related interference assesses the degree to which the pain affects the 7 domains of functioning (general activity, mood, walking ability, normal work, relations with other people, sleep and the enjoyment of life).

### 2.4 Psychological profile assessment

#### 2.4.1 Depression

The Beck's Depression Inventory (BDI‐II)^26^ is a self‐administered test used, in clinical practice and research, to assess depression. It is composed of 21 items, each scored from 0 to 3, with higher scores indicating higher levels of depression.

#### 2.4.2 Anxiety

The State–Trait Anxiety Inventory (STAI)^27^ is a tool commonly used to measure anxiety via the self‐reporting, grading and quantising of these symptoms. This type of test has 40 items, which are divided into 2 subscales, with 20 items each, termed respectively: The State Anxiety Scale (S‐Anxiety) and Trait Anxiety Scale (T‐Anxiety). First, the S‐STAI measures the state of anxiety during the administration period (instantly); secondly, the T‐STAI investigates the general aspects of anxious tendencies.^28^

#### 2.4.3 Sleep

Subjective sleep quality and daytime sleepiness were evaluated using the Pittsburgh Sleep Quality Index (PSQI)^29^ and Epworth Sleepiness Scale (ESS),^30^ respectively. First, the PSQI evaluates sleep quality, considering a period of 1 month, and its total score (0–21) is divided into 7 components (0–3), which are: subjective sleep quality, sleep latency, sleep duration, habitual sleep efficiency, sleep disturbances, use of sleeping medication and daytime dysfunction. Secondly, the ESS measures daytime sleepiness through 8 items, each scored from 0 to 3. In this scale a higher score corresponds to a higher sleep propensity in daily life.

### 2.5 Quality of life assessment

#### 2.5.1 Health‐related quality of life

The 36‐Item Short‐Form Health Survey (SF‐36)^31^ is the most frequently used instrument for the evaluation of the health‐related quality of life (HRQoL). The SF‐36 measures eight scales: physical functioning (PF), physical role (RP), bodily pain (BP), general health (GH), vitality (VT), social functioning (SF), emotional role (RE) and mental health (MH). The scores are transformed to range from zero, where the respondent has the worst possible health, to 100, where the respondent is in the best possible health.

#### 2.5.2 Oral health‐related quality of life

The OHIP‐14^32^ is a questionnaire composed of 14‐items used to evaluate the consequences of oral diseases in seven dimensions: functional limitations, social handicap, disability, physical disability, psychological disability, discomfort and pain.

### 2.6 Statistical analysis

The R software (v. 4.1 2) (Team Rcore, 2016) was used to perform the statistical analyses. Descriptive statistics, including means, standard deviations (SD), medians and interquartile ranges (IQR), were measured to analyse the socio‐demographic and clinical characteristics of the two groups. To assess the significant differences between the percentages in the two groups, Fisher's exact test was used.

The Mann–Whitney *U*‐test was performed to test any differences between the clinical parameters, psychological profile, sleep and QoL and to evaluate the recorded medians of the VAS, BPI, PD‐Q, SF‐MPQ, S‐STAI, T‐STAI, BDI, PSQI, ESS, SF‐36 and OHIP‐14. A dependency analysis among the pain tests and qualitative predictors in the BMS patients was computed. The Mann–Whitney *U*‐test was used to measure the dependency between the pain tests and gender, marital status, employment status, smoking and alcohol consumption. Spearman's linear correlation analysis was tested between the pain tests (VAS, SF‐MPQ, PD‐Q, BPI severity and BPI interference) and qualitative predictors (age, years of education, BMI, sleep duration, disease onset, S‐STAI, T‐STAI, BDI, PSQI, ESS, SF‐36 and OHIP‐14. For these analyses, the significant difference between correlation coefficients was measured using the Bonferroni correction.

Correlation matrices, using the patient group data only, were constructed to identify potential covariates. Finally, multivariate linear regression analyses were computed by entering all the identified variables/predictors of a univariate analysis; unadjusted coefficient estimations were obtained for each significant predictor identified from the correlation analysis. A total of nine models were computed. For each model, the adjusted *R* ^2^ is reported. It measures the overall goodness of fit adjusted for the number of variables included in the model.

The coefficient estimated for the binary variables (marital status, employment status, smoking and alcohol consumption) measures the effect of the Yes response on the outcome estimation. The demographic model (model 1) was computed to test the contribution of the demographic variables to the pain symptoms. Next, model 2 also considers the disease onset, model 3 the S‐STAI and T‐STAI, model 4 the BDI‐II, model 5 the sleep duration, model 6 the PSQI, model 7 the SF‐36 components and model 8 the OHIP. Each model was computed after controlling for demographic variables to test the contribution of the pain variables to the VAS, SF‐MPQ, PD‐Q, BPI severity and BPI interference, respectively.

Finally, a standard regression analysis (model 9) was computed by entering all the variables simultaneously into the model to estimate the relative contributions of all the variables to the pain symptoms. In all the steps, standard errors of the model coefficients, which measure the statistical precision of the inference estimation of the model parameters, are provided. The R software (v. 4.1 2) was used to conduct all the statistical analyses in this study, and a *p*‐value <.05 (two‐tails) was considered statistically significant. A post hoc power calculation was performed for the Mann – Whitney test. Considering the analysis of the different tests, the effect size ranged from 0.69 to 0.76 for a sample size of 40 participants in each group, with a significance level of 0.05. The power test value (1‐Beta) was from 0.91 to 0.97 (the analysis was performed with the Gpower software).

## 3 RESULTS

The socio‐demographic characteristics, the prevalence of systemic diseases and the drug consumption of the sample are shown in Table 1. No statistically significant differences were found between the BMS patients and controls in relation to gender, age, education level, family situation and employment status. However, the BMS patients reported a shorter sleep duration compared with the healthy subjects (6.04 ± 1.30; *p*‐value .020*) in addition a significantly higher proportion of the BMS patients were habitual moderate alcohol consumers compared to the controls (8 patients; 20%; *p*‐value: .029*). In addition, no statistically significant differences were found in relation to the prevalence of systemic diseases and drug consumption between the cases and controls.

**TABLE 1:** Socio‐demographic profile, risk factors, prevalence of systemic diseases and drug consumption of BMS patients and controls

	BMS	Controls	*p*‐Value
Demographic variables			
Gender	**Frequency (%)**	**Frequency (%)**	1.000
Male	10 (25%)	11 (27.5%)	
Female	30 (75%)	29 (72.5%)	
Age (in years)	**Mean ± SD**	**Mean ± SD**	.285
	65.6 ± 8.60	63.7 ± 7.10	
Education (in years)	9.3 ± 5.30	9.55 ± 5.29	.833
Family situation	**Frequency (%)**	**Frequency (%)**	.248
Single	3 (7.5%)	0 (0%)	
Married	33 (82.5%)	38 (95%)	
Divorced	1 (2.5%)	1 (2.5%)	
Widowed	3 (7.5%)	1 (2.5%)	
Employment	**Frequency (%)**	**Frequency (%)**	.205
Employed	8 (20%)	15 (37.5%)	
Unemployed	20 (50%)	17 (42.5%)	
Retired	12 (30%)	8 (20%)	
Body Mass Index (BMI)	**Mean ± SD**	**Mean ± SD**	.461
	27.5 ± 4.28	26.9 ± 3.50	
Sleep duration (h)	6.04 ± 1.30	6.68 ± 1.10	.020*
Risk factors			
Smoking	**Frequency (%)**	**Frequency (%)**	.386
Never smokers	30 (75%)	32 (80%)	
Very light smokers (<5 cigarettes)	4 (10%)	1 (2.5%)	
Light smokers (5–10 cigarettes)	2 (5%)	0 (0%)	
Moderate smokers (10–15 cigarettes)	2 (5%)	3 (7.5%)	
Heavy smokers (>15 cigarettes)	2 (5%)	4 (10%)	
Alcohol use	**Frequency (%)**	**Frequency (%)**	.029*
Moderate drinkers (<14 units/week)	8 (20%)	1 (2.5%)	
Not	32 (80%)	39 (97.5%)	
Systemic diseases			
Essential hypertension	19 (47.5%)	14 (35%)	.364
Hypercholesterolemia	14 (35%)	10 (25%)	.465
Myocardial infarction	0 (0%)	3 (7.5%)	.241
Asthma	2 (5%)	3 (7.5%)	1.000
Gastroesophageal reflux disease	8 (20%)	5 (12.5%)	.546
Endocrine disease	1 (2.5%)	0 (0%)	1.000
Thyroid disease	7 (17.5%)	6 (15%)	1.000
Benign prostatic hypertrophy	1 (2.5%)	1 (2.5%)	1.000
Drug consumption			
Beta blockers	7 (17.5%)	9 (22.5%)	.781
Angiotensin receptor blockers	3 (7.5%)	5 (12.5%)	.456
Diuretics	4 (10%)	7 (17.5%)	.518
Calcium channel blockers	4 (10%)	3 (7.5%)	1.000
ACE‐inhibitors	7 (17.5%)	4 (10%)	.518
Simvastatin	12 (30%)	6 (15%)	.180
Antiplatelets	9 (22.5%)	8 (20%)	1.000
Blood thinners	1 (2.5%)	2 (5%)	1.000
Levothyroxine sodium	1 (2.5%)	5 (12.5%)	.201
Proton pump inhibitors	13 (32.5%)	5 (12.5%)	.059

*Note:* The Significance difference between the percentages was measured by the Fisher's exact test. The Significance difference between means was measured by the T‐ test.

Abbreviation: BMS, burning mouth syndrome.

*Significant .01 < *p* ≤ .05.

Comparisons of the clinical parameters between the BMS patients and controls are summarised in Table 2. Considering the VAS scale, the patients with BMS reported a score median of 10 [8–10]; specifically, 2 patients (5%) had mild pain, 1 (2.5%) moderate pain and 37 (92.5%) severe pain. The PD‐Q scores showed a median of 8 [4.75–11.2]; precisely, 30 BMS patients (75%) had a PD‐Q total score < 12, 7 patients (17.5%) had a score between 12–19 and only 3 patients (7.5%) had a total score > 19. In contrast, in the healthy subject group all the patients (40; 100%) had a PD‐Q score < 12.

**TABLE 2:** Clinical parameters: Psychological profile, sleep and quality of life in BMS patients and controls

Clinical parameters	BMS (Median; IQR)	Controls (Median; IQR)	*p*‐Value
VAS	10 [8–10]	—	/
SF‐MPQ	5 [3–7.25]	—	/
PD‐Q	8 [4.75–11.2]	—	/
BPI			
Severity score	30 [19.8–37]	—	/
Interference score	18 [9–32]		
STAI state	50 [44.8–61]	37 [33–50]	<.001**
STAI trait	50 [43.8–55.2]	40 [33.8–48.2]	<.001**
BDI‐II	15 [10–23]	6 [2–9.25]	<.001**
PSQI	8.50 [4.75–11]	5 [3–7]	<.001**
Subjective sleep quality	1.50 [1–3]	1 [0.75–1]	<.001**
Sleep latency	2 [1–2]	1 [0–1]	<.001**
Sleep duration	1 [0.75–2]	1 [0–1]	.118
Habitual sleep efficiency	1 [0–1]	0 [0–1]	.233
Sleep disturbances	1 [0–2]	1 [1–1]	.992
Use of sleeping medication	0 [0–1]	0 [0–0]	<.001**
Daytime dysfunction	1 [0–1]	0.50 [0–1]	.072
ESS	5 [3–7.25]	4 [3–6]	.149
SF‐36			
Physical functioning (PF)	60 [43.8–100]	95 [75–100]	.007
Role physical (RP)	75 [0–100]	100 [68.8–100]	.027
Bodily pain (BP)	51 [40.8–61]	75 [52–100]	<.001**
General health (GH)	47 [36.5–57]	65 [45–72]	.008
Vitality (VT)	50 [35–50]	60 [47.5–85]	<.001**
Social functioning (SF)	62 [46.8–75]	87 [75–90.2]	<.001**
Role emotional (RE)	66 [0–100]	100 [66–100]	.002**
Mental health (MH)	48 [40–57]	72 [60–80]	<.001**
OHIP‐14	21.5 [12–26]	4 [3–7.25]	<.001**

*Note:* The significance difference between medians was measured by the Mann–Whitney test.

Abbreviations: BDI, Beck Depression Inventory; BMS, burning mouth syndrome; BPI, brief pain inventory; ESS, Epworth Sleepiness Scale; IQR, interquartile range; OHIP‐14, Oral Health Impact Profile‐14; PD‐Q, PainDETECT Questionnaire; PSQI, Pittsburgh Sleep Quality Index; SF‐36, 36 items Short‐Form Survey; SF‐MPQ, Short‐form McGill Pain Questionnaire; STAI, State–Trait Anxiety Inventory; VAS, visual analogue scale.

**Significant with Bonferroni correction .002.

In addition, the BMS patients showed statistically significant differences in the medians and IQRs of the scores of the STAI and BDI‐II (*p*‐value <.001**). In detail, in the BMS group 37 patients (92.5%) and 34 patients (85%) had a S‐STAI and T‐STAI score higher than 40, respectively, while in the healthy group only 18 subjects (45%) and 22 subjects (55%) had these scores, respectively, such scores being greater than the predictive threshold value. These results suggest that the majority of patients with BMS are affected by anxiety, both trait and state. Considering the BDI‐II test, the median and IQR range of the scores in the BMS group were 15 and [10–23, respectively, while in the healthy subject group they were 6 and [2–9.25], respectively.

8 (20%) BMS patients did not show any clinical depression (score < 10) while 19 (47.5%), 10 (25%) and 3 (7.5%) patients presented mild depression (score: 10–18), moderate depression (score: 19–29) and severe depression (score > 30), respectively. Regarding sleep evaluation, the BMS patients showed a strongly statistically significant difference in the PSQI total score (*p* < .001**) and in the three component scores (subjective sleep quality, sleep latency and use of sleeping medication (*p*‐value <.001**). Poor sleep (PSQI > 5) was found in 30 BMS patients (75%) while only in 21 of the healthy subjects (52.5%). Although no statistically significant differences were found in the ESS total score between the two groups. Statistically significant differences were found in the median and IQR of the SF‐36 sub‐items (BP, *p* < .001**; VT, *p* < .001**; SF, <0.001**; RE, 0.002**; MH, <0.001**) and in the total score of OHIP‐14 (*p* < .001**) between the patients and controls, suggesting an impairment in the HRQoL and Oral health‐related quality of life (OHQoL) of the BMS patients.

The type and location of the oral symptoms are shown in Table 3. Statistically significant differences were found between the cases and controls in relation to most of the symptoms. All the BMS patients reported a burning sensation followed by xerostomia (30; 75%), a change in the tongue morphology (25; 62.5%) and dysgeusia (19; 47.5%). The worst symptoms reported by the BMS patients were burning (57.5%) followed by xerostomia (17.5%) and a change in the tongue morphology (10%).

**TABLE 3:** Prevalence of oral symptoms and sites involved in BMS patients and control subjects; Disease onset, number of consulted doctors and typology of referrals and worst symptom in BMS patients

	BMS frequency (%)	Controls frequency (%)	*p*‐Value
Oral symptoms			
Burning	40 (100%)	0	<.001**
Xerostomia	30 (75%)	2 (5%)	<.001**
Dysgeusia	19 (47.5%)	0	<.001**
Sialorrhea	7 (17.5%)	0	.012*
Globus pharyngeus	15 (37.5%)	0	<.001**
Itching	5 (12.5%)	0	.055
Intra‐oral foreign body sensation	10 (25%)	0	.001**
Tingling sensation	11 (27.5%)	0	<.001**
Occlusal dysesthesia	6 (15%)	0	.026
Change in tongue morphology	25 (62.5%)	0	<.001**
Oral dyskinesia	3 (7.5%)	0	.241
Dysosmia	2 (5%)	0	.494
Location of pain/burning			
Gums	26 (65%)	0	<.001**
Cheeks	26 (65%)	1 (2.5%)	<.001**
Lips	33 (82.5%)	1 (2.5%)	<.001**
Tongue	38 (95%)	0	<.001**
Floor of the mouth	21 (52.5%)	0	<.001**
Anterior palate	25 (62.5%)	0	<.001**
Soft palate	18 (45%)	0	<.001**
	**BMS**		
Disease onset in BMS patients (months)	*Mean ± SD* 21.40 ± 25.25		
Number of doctors consulted prior to diagnosis of BMS	*Median; IQR* 2; [2, 3]		
Referrals	*Frequency (%)*		
Dentist	31 (77.5%)		
Physician	22 (55%)		
Maxillofacial surgeon	7 (17.5%)		
Otolaryngologist	8 (20%)		
Gastroenterologist	10 (25%)		
Dermatologist	2 (5%)		
Neurologist	4 (10%)		
Psychiatrist	1 (2.5%)		
Others	6 (15%)		
Self‐reported cause of disease	*Frequency (%)*		
No attribution	11 (27.5%)		
After dental practice	14 (35%)		
After stressful life event	12 (30%)		
After systemic infection	3 (7.5%)		
Worst symptom	*Frequency (%)*		
Burning	23 (57.5%)		
Xerostomia	7 (17.5%)		
Change in tongue morphology	4 (10%)		
Globus	1 (2.5%)		
Dysgeusia	3 (7.5%)		
Sialorrhea	2 (5%)		

*Note:* The Significance difference between the percentages was measured by the Fisher's exact test. The significance difference between medians was measured by the Mann–Whitney test.

Abbreviations: BMS, burning mouth syndrome; IQR, interquartile range.

**Significant with Bonferroni correction .004.

In addition, the BMS patients had consulted an average of two specialists each and had waited 21.40 ± 25.25 months before obtaining a proper diagnosis. Dentists (31; 77.5%) and physicians (22; 55%) were the most frequent doctors consulted. Regarding the causes of disease attributed by the patients; in the majority of cases the onset of the BMS symptomatology appeared after dental treatment (14; 35%) or after a stressful life event (12; 30%).

The linear correlation analysis between the VAS, SF‐MPQ, PD‐Q, BPI severity and BPI interference and the quantitative and qualitative predictors in the BMS patient group is summarised in Table 4. Specifically, among the quantitative predictors a statistically significant positive correlation was found between the patient's age and BPI interference (*p*‐value: .031*) and between sleep duration and VAS, SF‐MPQ and BPI severity and interference (*p*‐value: .011*; .035*; .023*; .044*, respectively). A strongly statistically positive correlation was found between disease onset, S‐STAI, BDI‐II with all the pain questionnaires (*p*‐value: <.001**) and between T‐ STAI with VAS, SF‐MPQ, PD‐Q and BPI severity and interference (*p*‐value: .002**; <.001**;<.001**;<.001**, .002**, respectively). A positive correlation was found between PSQI and VAS, SF‐MPQ, BPI severity and BPI intensity (*p*‐value:<.001**; .008**; <.001**; <.001**, respectively) while ESS correlates only with PD‐Q (*p*‐value: .041*) and BPI severity (*p*‐value: .013*). In addition, a strongly statistically positive correlation was found between every component of the SF‐36 and OHIP‐14 total score and all the pain questionnaires. Evaluating the qualitative predictors there is a significant positive correlation only between marital status and the VAS scale (*p*‐value: .025*).

**TABLE 4:** Linear Correlation analysis between pain tests and quantitative/qualitative predictors

Predictors\Pain tests	VAS		SF‐MPQ		PD‐Q		BPI severity		BPI interference	
Quantitative predictors	*ρ*	*p*‐Value	*ρ*	*p*‐Value	*ρ*	*p*‐Value	*ρ*	*p*‐Value	*ρ*	*p*‐Value
Age	.018	.109	.052	.646	.088	.437	.179	.113	.18	.031*
Education	−.067	.553	.036	.752	.094	.407	−.048	.672	−.067	.158
BMI	.137	.227	.009	.939	.005	.963	.019	.869	.137	.989
Sleep duration	−.281	.011*	−.236	.035*	−.179	.112	−.255	.023*	−.281	.044*
Disease onset	.888	<.001**	.747	<.001**	.769	<.001**	.606	<.001**	.888	<.001**
STAI state	.548	<.001**	.446	<.001**	.497	<.001**	.481	<.001**	.548	<.001**
STAI trait	.337	.002**	.407	<.001**	.415	<.001**	.365	<.001**	.337	.002**
BDI‐II	.517	<.001**	.596	<.001**	.648	<.001**	.668	<.001**	.517	<.001**
PSQI	.409	<.001**	.296	.008**	.21	.061	.485	<.001**	.409	<.001**
ESS	.169	.134	.195	.083	.229	.041*	.278	.013*	.169	.054
SF‐36 PF	−.317	.004**	−.3	.007**	−.398	<.001**	−.395	<.001**	−.317	<.001**
SF‐36 RP	−.243	.03*	−.315	.004**	−.308	.005**	−.348	.002**	−.243	<.001**
SF‐36 BP	−.44	<.001**	−.455	<.001**	−.478	<.001**	−.454	<.001**	−.44	<.001**
SF‐36 GH	−.287	.01*	−.316	.004**	−.37	.001**	−.406	<.001**	−.287	<.001**
SF‐36 VT	−.38	<.001**	−.374	<.001**	−.417	<.001**	−.578	<.001**	−.38	<.001**
SF‐36 SF	−.366	<.001**	−.439	<.001**	−.509	<.001**	−.529	<.001**	−.366	<.001**
SF‐36 RE	−.381	<.001**	−.373	<.001**	−.395	<.001**	−.509	<.001**	−.381	<.001**
SF‐36 MH	−.549	<.001**	−.525	<.001**	−.548	<.001**	−.645	<.001**	−.549	<.001**
OHIP‐14	.677	<.001**	.627	<.001**	.651	<.001**	.604	<.001**	.677	<.001**

	VAS		SF‐MPQ		PD‐Q		BPI severity BPI interference			
Qualitative predictors	Median [Q1:Q3]	*p*‐Value	Median [Q1:Q3]	*p*‐Value	Median [Q1:Q3]	*p*‐Value	Median [Q1:Q3]	*p*‐Value	Median [Q1:Q3]	*p*‐Value
Gender										
Female	10 [8.25–10]	.296	5 [3–8.5]	.718	8.5 [4.25–11]	.876	30 [19.2–35.8]	.547	18 [9.25–31.8]	.863
Male	8 [8–10]		6 [5–6.75]		8 [6.5–11.5]		30 [30–38]		19 [9.5–31.5]	
Marital status										
Married	10 [7–10]	.025*	5 [3–7]	.497	8 [4–11]	.803	30 [11–35]	.176	17 [7–32]	.433
Not married	10 [10–10]		6 [5–8]		8 [8–11]		30 [30–40]		22 [14.5–34.5]	
Employment status										
Employed	10 [9.5–10]	.410	6.5 [5–11]	.089	8.5 [7.5–9.5]	.599	19.5 [10–40]	.440	18 [12.8–30.5]	.959
Not employed	10 [7.75–10]		5 [3–6.25]		8 [4–12]		30 [23–35.2]		18 [9–32]	
Smoking status										
Smoker	9.5 [7.25–10]	.513	5.5 [5–6]	.814	9.5 [8–11]	.471	30 [28.5–37.8]	.635	19 [17–25]	.925
Non‐smoker	10 [8–10]		5 [3–7.75]		8 [4.25–11.8]		30 [19.2–35.8]		16.5 [7.5–32.8]	
Alcohol use										
Yes	10 [8.75–10]	.789	5.5 [3.7–6.2]	.825	7 [4–8.75]	.351	29 [10.2–30.2]	.391	18 [14.5–31.2]	.946
No	10 [7.75–10]		5 [3–8.25]		9 [5–12]		30 [20–40]		18 [9–32.2]	

*Note: ρ* is Spearman's correlation coefficient; Median [First Quartile Q1; Third Quartile Q3]. Wilcoxon‐Mann–Whitney Test.

Abbreviations: BDI, Beck's Depression Inventory; BMI, Body Mass Index; BMS, burning mouth syndrome; BP, bodily pain; BPI, brief pain inventory; ESS, Epworth Sleepiness Scale; GH, general health; MH, mental health; OHIP‐14, Oral Health Impact Profile‐14; PD‐Q, PainDETECT Questionnaire; PF, physical function; PSQI, Pittsburgh Sleep Quality Index; RE, role emotional; RP, role physical; SF, social functioning; SF‐36, 36 items Short‐Form Survey; SF‐MPQ, Short‐form McGill Pain Questionnaire; STAI, State–Trait Anxiety Inventory; VAS, visual analogue scale; VT, vitality.

*p*‐Value: *Significant .01 < *p*‐Value ≤ .05. **Significant *p*‐Value ≤ .01.

The results of the simultaneous multiple linear regression analyses for the BMS patients, predicting the VAS, SF‐MPQ, PD‐Q, BPI severity and BPI intensity, are summarised in Table 5. In detail, no predictors were found to correlate with VAS in the BMS patients. In the second model (SF‐MPQ), the contribution of T‐STAI, BDI‐II and OHIP‐14 was found to be positively correlated with SF‐MPQ in the BMS patients (*β*: 0.44 *p*‐value: .016*; *β*: 0.50 *p*‐value: < .001**; and *β*: 0.52 *p*‐value: < .001**, respectively) resulting in a significant increase in the *R* ^2^ value (DR2 = 21.8% *p*‐value: .008**; DR2 = 32.1% *p*‐value < .001**; and DR2 = 41.3 *p*‐value < .001**, respectively). In the third model (PD‐Q); the contribution of BDI‐II and OHIP‐14 was found to be positively correlated with PD‐Q in the BMS patients (*β*: 0.34 *p*‐value: .004**; and *β*: 0.33 *p*‐value: .003**, respectively) resulting in a significant increase in the *R* ^2^ value (DR2 = 13.3% *p*‐value: .004**; and DR2 = 13.8% *p*‐value: .003**; respectively). In the fourth model (BPI severity) the contribution of BDI‐II, ESS and OHIP‐14 was found to be positively correlated with BPI severity in the BMS patients (*β*: 0.70 *p*‐value: .004**; *β*: 1.92 *p*‐value: .004**; and *β*: 0.49 *p*‐value: .037*, respectively) resulting in a significant increase in the *R* ^2^ value (DR2 = 16.5% *p*‐value < .004**; DR2 = 21.1% *p*‐value .004**; and DR2: 4.4% *p*‐value: .037*, respectively). In the fifth model (BPI interference) the contribution of BDI‐II, PSQI and OHIP‐14 was found to be positively correlated with BPI interference in the BMS patients (*β*: 1.19 *p*‐value < .001**; *β*: 1.61 *p*‐value: .0144*; and *β*: 1.08 *p*‐value < .001**, respectively) resulting in a significant increase in the *R* ^2^ value (DR2 = 31.2% *p*‐value < .001**; DR2 = 5.7% *p*‐value .014*; and DR2: 28.0% *p*‐value < .001**, respectively).

**TABLE 5:** Multiple linear regression model predicting VAS, SF‐MPQ, PD‐Q, BPI severity and BPI interference in BMS patients

Multiple linear regression model predicting VAS																		
Predictors	Model 1		Model 2		Model 3		Model 4		Model 5		Model 6		Model 7		Model 8		Model 9	
	*β* (SE)	*p*‐Value	*β* (SE)	*p*‐Value	*β* (SE)	*p*‐Value	*β* (SE)	*p*‐Value	*β* (SE)	*p*‐Value	*β* (SE)	*p*‐Value	*β* (SE)	*p*‐Value	*β* (SE)	*p*‐Value	*β* (SE)	*p*‐Value
Age	0.02 (0.04)	.572	0.02 (0.04)	.621	0.03 (0.04)	.445	0.03 (0.04)	.557	0.02 (0.04)	.668	0.02 (0.04)	.700	−0.00 (0.05)	.971	0.03 (0.04)	.426	0.02 (0.05)	.622
Education	−0.01 (0.07)	.926	−0.02 (0.07)	.800	0.03 (0.07)	.648	−0.00 (0.07)	.961	−0.01 (0.07)	.832	−0.00 (0.06)	.990	−0.05 (0.07)	.538	0.00 (0.06)	.977	−0.00 (0.08)	.977
Gender: Female	0.86 (0.79)	.281	0.83 (0.81)	.312	0.71 (0.79)	.380	0.92 (0.82)	.272	0.96 (0.81)	.246	1.02 (0.8)	.210	1.33 (0.88)	.146	0.70 (0.78)	.381	1.57 (0.97)	.127
Marital status: Married	−1.79 (0.94)	.065	−1.80 (0.95)	.067	−1.80 (0.94)	.066	−1.78 (0.95)	.071	−1.69 (0.95)	.084	−1.70 (0.93)	.076	−0.83 (1.08)	.452	−1.57 (0.92)	.097	−0.77 (1.18)	.524
Employment status: Employed	0.88 (0.79)	.277	0.78 (0.82)	.349	0.69 (0.79)	.388	0.85 (0.81)	.300	0.83 (0.80)	.309	0.88 (0.78)	.273	1.29 (0.84)	.137	0.63 (0.78)	.431	0.61 (0.91)	.508
Smoking status: Smoker	−0.46 (0.78)	.558	−0.33 (0.8)	.687	−0.86 (0.78)	.282	−0.44 (0.78)	.578	−0.37 (0.78)	.642	−0.38 0.76	.618	−1.08 (0.85)	.220	−0.43 (0.74)	.565	−1.52 (0.97)	.136
Alcohol use (>14 units/week)	−0.15 (0.8)	.855	−0.04 (0.82)	.959	−0.21 (0.78)	.793	−0.09 (0.82)	.904	−0.07 (0.81)	.933	−0.17 (0.79)	.832	0.25 (0.91)	.786	0.15 (0.79)	.851	0.36 (1.05)	.733
Body Mass Index (BMI)	0.04 (0.08)	.614	0.05 (0.08)	.554	0.05 (0.08)	.531	0.04 (0.08)	.621	0.04 (0.08)	.648	0.04 (0.08)	.589	−0.01 (0.1)	.918	0.04 (0.08)	.617	−0.06 (0.11)	.604
Disease onset			0.01 (0.01)	.524													0.00 (0.02)	.955
STAI state					0.08 (0.05)	.092											0.12 (0.05)	.036*
STAI trait					−0.03 (0.05)	.481											−0.05 (0.06)	.442
BDI‐II							0.01 (0.04)	.719									0.06 (0.07)	.418
Sleep duration (h)									−0.19 (0.25)	.452							0.02 (0.5)	.965
PSQI											0.1 (0.07)	.188					0.09 (0.15)	.555
SF‐36 PF													−0.01 (0.01)	.505			−0.01 (0.02)	.565
SF‐36 RP													0.01 (0.01)	.655			0.01 (0.01)	.664
SF‐36 BP													−0.02 (0.02)	.425			−0.02 (0.03)	.455
SF‐36 GH													0.00 (0.03)	.951			0.03 (0.03)	.441
SF‐36 VT													0.02 (0.05)	.636			0.00 (0.05)	.935
SF‐36 SF													0.04 (0.02)	.076			0.05 (0.02)	.059
SF‐36 RE													−0.03 (0.01)	.063			−0.02 (0.02)	.150
SF‐36 MH													−0.01 (0.04)	.836			0.06 (0.05)	.284
OHIP‐14															0.06 (0.03)	.094	0.04 (0.05)	.486
*R* ^2^ (%)	.0	.506	.0	.572	3.4	.034*	.0	.604	.0	.554	1.1	.426	.0	.540	4.7	.324	3.3	.464
*R* ^2^ change (%)			.0	.524	3.4	.186	.0	.719	.0	.452	1.1	.188	.0	.488	4.7	.094	3.3	.424

Multiple linear regression model predicting SF‐MPQ																		
Predictors	Model 1		Model 2		Model 3		Model 4		Model 5		Model 6		Model 7		Model 8		Model 9	
	*β* (SE)	*p*‐Value	*β* (SE)	*p*‐Value	*β* (SE)	*p*‐Value	*β* (SE)	*p*‐Value	*β* (SE)	*p*‐Value	*β* (SE)	*p*‐Value	*β* (SE)	*p*‐Value	*β* (SE)	*p*‐Value	*β* (SE)	*p*‐Value
Age (in years)	−0.00 (0.17)	.981	−0.01 (0.17)	.935	−0.07 (0.15)	.659	0.05 (0.14)	.745	0.00 (0.17)	.988	−0.02 (0.17)	.888	−0.08 (0.18)	.665	0.08 (0.13)	.557	0.05 (0.16)	.755
Education (in years)	0.50 (0.27)	.073	0.46 (0.28)	.116	0.55 (0.25)	.034*	0.60 (0.22)	.011*	0.51 (0.28)	.076	0.51 (0.27)	.067	0.57 (0.27)	.047*	0.57 (0.21)	.009**	0.43 (0.25)	.112
Gender: Female	2.71 (3.33)	.422	2.55 (3.38)	.457	3.53 (2.96)	.242	4.17 (2.74)	.139	2.60 (3.42)	.452	3.10 (3.38)	.366	0.96 (3.33)	.775	1.05 (2.55)	.684	0.39 (3.21)	.905
Marital status: Married	−2.88 (3.91)	.466	−2.90 (3.95)	.469	−5.65 (3.53)	.120	−2.43 (3.19)	.452	−3.00 (4.00)	.459	−2.64 (3.93)	.508	−1.26 (4.09)	.761	−0.84 (2.99)	.781	−2.69 (3.90)	.501
Employment status: Employed	4.77 (3.32)	.160	4.38 (3.41)	.209	5.61 (2.95)	.067	3.79 (2.71)	.173	4.83 (3.38)	.163	4.76 (3.33)	.163	5.02 (3.17)	.127	2.43 (2.56)	.350	1.97 (2.99)	.520
Smoking status: Smoker	−3.30 (3.20)	.310	−2.81 (3.34)	.408	−4.61 (2.92)	.125	−2.69 (2.62)	.312	−3.41 (3.29)	.307	−3.11 (3.22)	.343	−2.93 (3.23)	.373	−3.11 (2.43)	.211	−0.90 (3.18)	.780
Alcohol use (>14 units/week)	1.22 (3.32)	.716	1.62 (3.42)	.640	0.29 (2.92)	.923	3.00 (2.74)	.282	1.12 (3.40)	.745	1.16 (3.33)	.731	2.19 (3.45)	.532	3.95 (2.58)	.136	6.39 (3.44)	.082
Body Mass Index (BMI)	0.1 (0.34)	.780	0.13 (0.35)	.716	0.05 (0.3)	.875	0.09 (0.28)	.754	0.1 (0.35)	.774	0.10 (0.34)	.766	0.37 (0.39)	.359	0.08 (0.26)	.755	0.23 (0.37)	.540
Disease onset (in months)			0.03 (0.06)	.558													0.08 (0.05)	.175
STAI state					−0.04 (0.17)	.809											−0.14 (0.18)	.438
STAI trait					0.44 (0.17)	.016*											0.29 (0.21)	.191
BDI‐II							0.50 (0.12)	<.001**									0.06 (0.22)	.802
Sleep duration (h)									0.24 (1.05)	.819							−1.56 (1.63)	.352
PSQI											0.26 (0.30)	.394					−0.57 (0.49)	.263
SF‐36 PF													0.05 (0.05)	.391			0.04 (0.05)	.484
SF‐36 RP													−0.05 (0.04)	.231			−0.07 (0.04)	.103
SF‐36 BP													−0.05 (0.09)	.586			0.03 (0.08)	.748
SF‐36 GH													0.02 (0.1)	.826			0.02 (0.11)	.874
SF‐36 VT													0.11 (0.17)	.534			0.08 (0.15)	.597
SF‐36 SF													−0.03 (0.08)	.682			−0.00 (0.08)	.964
SF‐36 RE													−0.03 (0.05)	.532			0.02 (0.05)	.662
SF‐36 MH													−0.22 (0.16)	.192			−0.17 (0.16)	.304
OHIP‐14															0.52 (0.11)	<.001**	0.51 (0.17)	.009**
*R* ^2^ (%)	.0	.529	.0	.601	21.8	.060	32.1	.010*	.0	.635	.0	.557	15.4	.206	41.3	.001**	39.4	.065
*R* ^2^ change (%)			.0	.558	21.8	.008**	32.1	<.001**	.0	.819	.0	.394	15.4	.128	41.3	<.001**	39.4	.045*

Multiple linear regression model predicting PD‐Q																		
Predictors	Model 1		Model 2		Model 3		Model 4		Model 5		Model 6		Model 7		Model 8		Model 9	
	*β* (SE)	*p*‐Value	*β* (SE)	*p*‐Value	*β* (SE)	*p*‐Value	*β* (SE)	*p*‐Value	*β* (SE)	*p*‐Value	*β* (SE)	*p*‐Value	*β* (SE)	*p*‐Value	*β* (SE)	*p*‐Value	*β* (SE)	*p*‐Value
Age (in years)	−0.06 (0.14)	.690	−0.06 (0.14)	.669	−0.07 (0.14)	.602	−0.02 (0.14)	.859	−0.02 (0.14)	.889	−0.05 (0.15)	.711	−0.12 (0.15)	.448	−0.01 (0.12)	.963	−0.01 (0.17)	.951
Education (in years)	0.22 (0.22)	.328	0.20 (0.23)	.402	0.28 (0.23)	.234	0.29 (0.2)	.150	0.28 (0.22)	.217	0.22 (0.24)	.366	0.27 (0.24)	.263	0.27 (0.2)	.185	0.38 (0.29)	.215
Gender: Female	0.48 (2.76)	.863	0.39 (2.81)	.890	0.64 (2.75)	.818	1.49 (2.45)	.549	−0.13 (2.72)	.963	0.36 (3.09)	.907	−0.92 (2.90)	.753	−0.56 (2.45)	.820	−0.41 (4.39)	.928
Marital status: Married	−1.66 (3.24)	.611	−1.67 (3.28)	.614	−2.81 (3.28)	.399	−1.35 (2.85)	.640	−2.32 (3.18)	.471	−1.66 (3.29)	.617	−2.46 (3.57)	.497	−0.39 (2.87)	.894	−0.66 (4.15)	.876
Employment status: Employed	1.96 (2.74)	.480	1.76 (2.84)	.539	2.12 (2.74)	.447	1.29 (2.43)	.600	2.30 (2.69)	.399	1.86 (3.03)	.543	1.77 (2.76)	.527	0.50 (2.46)	.839	1.34 (3.97)	.740
Smoking status: Smoker	−0.04 (2.65)	.989	0.22 (2.78)	.936	−0.99 (2.71)	.719	0.39 (2.34)	.869	−0.65 (2.62)	.805	0.03 (2.80)	.991	0.26 (2.81)	.926	0.09 (2.33)	.970	−0.64 (3.61)	.861
Alcohol use (>14 units/week)	−2.76 (2.75)	.322	−2.56 (2.84)	.375	−3.21 (2.71)	.246	−1.53 (2.45)	.537	−3.32 (2.70)	.229	−2.73 (2.82)	.339	−3.12 (3.01)	.310	−1.06 (2.47)	.672	−2.26 (3.38)	.513
Body Mass Index (BMI)	−0.12 (0.28)	.682	−0.10 (0.29)	.732	−0.13 (0.28)	.651	−0.12 (0.25)	.626	−0.09 (0.28)	.743	−0.11 (0.3)	.718	−0.04 (0.34)	.909	−0.13 (0.25)	.615	−0.10 (0.41)	.804
Disease onset (in months)			0.02 (0.05)	.712													−0.01 (0.05)	.933
STAI state					0.07 (0.16)	.686											0.06 (0.21)	.788
STAI trait					0.15 (0.16)	.363											−0.16 (0.21)	.451
BDI‐II							0.34 (0.11)	.004**									0.24 (0.24)	.336
Sleep duration (h)									1.34 (0.84)	.120							1.51 (0.98)	.142
ESS											0.03 (0.35)	.931					−0.13 (0.64)	.837
SF‐36 PF													−0.00 (0.05)	.959			−0.00 (0.05)	.969
SF‐36 RP													−0.03 (0.04)	.448			−0.02 (0.05)	.709
SF‐36 BP													−0.05 (0.08)	.533			−0.06 (0.09)	.523
SF‐36 GH													−0.01 (0.09)	.915			0.01 (0.11)	.914
SF‐36 VT													0.05 (0.15)	.743			0.02 (0.16)	.922
SF‐36 SF													−0.10 (0.07)	.187			−0.04 (0.08)	.583
SF‐36 RE													0.03 (0.04)	.495			0.01 (0.07)	.899
SF‐36 MH													−0.05 (0.14)	.734			−0.01 (0.16)	.963
OHIP‐14															0.33 (0.10)	.003**	0.12 (0.18)	.514
*R* ^2^ (%)	.0	.851	.0	.901	.0	.703	13.3	.141	.0	.665	.0	.911	.0	.536	13.8	.134	.0	.574
*R* ^2^ change (%)			.0	.713	.0	.232	13.3	.004**	.0	.120	.0	.931	.0	.268	13.8	.003**	.0	.396

Multiple linear regression model predicting BPI severity in BMS patients																		
Predictors	Model 1		Model 2		Model 3		Model 4		Model 5		Model 6		Model 7		Model 8		Model 9	
	*β* (SE)	*p*‐Value	*β* (SE)	*p*‐Value	*β* (SE)	*p*‐Value	*β* (SE)	*p*‐Value	*β* (SE)	*p*‐Value	*β* (SE)	*p*‐Value	*β* (SE)	*p*‐Value	*β* (SE)	*p*‐Value	*β* (SE)	*p*‐Value
Age (in years)	−0.13 (0.29)	.662	−0.17 (0.29)	.567	−0.22 (0.28)	.438	−0.06 (0.26)	.825	−0.14 (0.3)	.638	−0.03 (0.25)	.896	−0.09 (0.29)	.759	−0.05 (0.27)	.856	0.22 (0.25)	.399
Education (in years)	−0.59 (0.46)	.210	−0.75 (0.47)	.119	−0.62 (0.46)	.191	−0.44 (0.41)	.287	−0.61 (0.47)	.207	−0.90 (0.41)	.036*	−0.67 (0.45)	.149	−0.52 (0.43)	.242	−1.23 (0.45)	.015*
Gender: Female	1.03 (5.68)	.857	0.40 (5.62)	.944	2.44 (5.56)	.665	3.10 (5.05)	.545	1.26 (5.82)	.830	−5.28 (5.46)	.341	3.20 (5.53)	.569	−0.55 (5.41)	.919	−1.46 (6.41)	.822
Marital status: Married	−6.74 (6.65)	.319	−6.80 (6.57)	.309	−10.02 (6.63)	.141	−6.10 (5.87)	.308	−6.49 (6.81)	.348	−6.17 (5.72)	.290	−0.46 (6.81)	.947	−4.81 (6.34)	.455	−4.51 (5.92)	.458
Employment status: Employed	−5.84 (5.65)	.309	−7.31 (5.67)	.207	−4.36 (5.55)	.439	−7.23 (5.00)	.159	−5.97 (5.75)	.308	−12.3 (5.26)	.026*	−4.67 (5.26)	.384	−8.06 (5.42)	.148	−14.1 (5.80)	.028*
Smoking status: Smoker	0.56 (5.45)	.919	2.45 (5.56)	.662	−0.00 (5.48)	.999	1.43 (4.82)	.769	0.79 (5.60)	.888	5.16 (4.86)	.297	0.62 (5.36)	.909	0.75 (5.15)	.886	9.99 (5.22)	.075
Alcohol use (>14 units/week)	−6.13 (5.65)	.286	−4.61 (5.68)	.423	−7.09 (5.48)	.206	−3.60 (5.05)	.481	−5.92 (5.78)	.314	−4.27 (4.89)	.389	−6.55 (5.74)	.266	−3.54 (5.46)	.522	−0.83 (5.17)	.874
Body Mass Index (BMI)	−0.23 (0.58)	.696	−0.11 (0.58)	.855	−0.31 (0.56)	.586	−0.24 (0.51)	.642	−0.24 (0.59)	.689	0.27 (0.52)	.610	−0.22 (0.65)	.738	−0.24 (0.55)	.661	−0.09 (0.58)	.883
Disease onset (in months)			0.13 (0.09)	.185													0.11 (0.08)	.213
STAI state					−0.25 (0.32)	.442											−0.61 (0.3)	.061
STAI trait					0.61 (0.32)	.068											0.39 (0.31)	.232
BDI‐II							0.70 (0.23)	.004**									0.51 (0.35)	.167
Sleep duration (h)									−0.51 (1.79)	.779							2.11 (2.41)	.396
PSQI											0.58 (0.45)	.201					0.87 (0.73)	.256
ESS											1.92 (0.61)	.004**					2.02 (0.92)	.043*
SF‐36 PF													−0.05 (0.09)	.547			−0.07 (0.08)	.404
SF‐36 RP													0.08 (0.07)	.259			0.02 (0.07)	.736
SF‐36 BP													0.23 (0.15)	.135			0.23 (0.13)	.096
SF‐36 GH													0.07 (0.17)	.676			0.14 (0.16)	.396
SF‐36 VT													−0.61 (0.29)	.046*			−0.52 (0.23)	.038*
SF‐36 SF													0.05 (0.13)	.740			0.10 (0.11)	.384
SF‐36 RE													−0.17 (0.08)	.048*			−0.03 (0.1)	.780
SF‐36 MH													0.17 (0.27)	.548			0.36 (0.24)	.163
OHIP‐14															0.49 (0.23)	0.037*	0.40 (0.29)	.187
*R* ^2^ (%)	.0	.714	.0	.606	.0	.472	16.5	.098	.0	.798	21.1	.065	15.4	.206	4.4	.331	52.1	.023*
*R* change (%)			.0	.185	.0	.141	16.5	.004**	.0	.779	21.1	.004**	15.4	.087	4.4	.037*	52.1	.011*

Multiple linear regression model predicting BPI interference in BMS patients																		
Predictors	Model 1		Model 2		Model 3		Model 4		Model 5		Model 6		Model 7		Model 8		Model 9	
	*β* (SE)	*p*‐Value	*β* (SE)	*p*‐Value	*β* (SE)	*p*‐Value	*β* (SE)	*p*‐Value	*β* (SE)	*p*‐Value	*β* (SE)	*p*‐Value	*β* (SE)	*p*‐Value	*β* (SE)	*p*‐Value	*β* (SE)	*p*‐Value
Age (in years)	−0.17 (0.38)	.650	−0.21 (0.38)	.591	−0.19 (0.39)	.627	−0.06 (0.30)	.853	−0.21 (0.39)	.595	−0.30 (0.35)	.401	−0.17 (0.41)	.677	−0.01 (0.31)	.983	0.12 (0.29)	.687
Education (in years)	−0.69 (0.60)	.259	−0.84 (0.63)	.191	−0.56 (0.64)	.389	−0.45 (0.48)	.355	−0.75 (0.62)	.236	−0.61 (0.56)	.283	−0.60 (0.63)	.350	−0.54 (0.49)	.271	−0.21 (0.46)	.648
Gender: Female	2.21 (7.47)	.769	1.65 (7.52)	.827	2.26 (7.66)	.770	5.69 (5.91)	.343	2.79 (7.63)	.717	4.61 (6.92)	.510	2.25 (7.76)	.774	−1.23 (6.05)	.840	4.79 (5.77)	.418
Marital status: Married	−1.60 (8.75)	.856	−1.65 (8.78)	.852	−3.43 (9.12)	.709	−0.52 (6.87)	.940	−0.97 (8.93)	.914	−0.09 (8.06)	.992	4.27 (9.55)	.659	2.61 (7.09)	.715	13.63 (7.01)	.070
Employment status: Employed	−1.50 (7.43)	.841	−2.80 (7.59)	.715	−1.49 (7.64)	.847	−3.84 (5.85)	.517	−1.83 (7.54)	.810	−1.57 (6.82)	.820	−2.20 (7.38)	.769	−6.33 (6.07)	.305	−6.97 (5.37)	.213
Smoking status: Smoker	−1.85 (7.17)	.799	−0.19 (7.44)	.980	−3.84 (7.55)	.615	−0.38 (5.64)	.946	−1.26 (7.34)	.865	−0.64 (6.6)	.923	−3.24 (7.52)	.671	−1.44 (5.76)	.805	0.46 (5.72)	.937
Alcohol use (>14 units/week)	−0.48 (7.43)	.949	0.85 (7.60)	.911	−1.25 (7.55)	.869	3.78 (5.91)	.527	0.05 (7.58)	.995	−0.84 (6.82)	.902	−3.79 (8.06)	.643	5.17 (6.11)	.404	1.43 (6.19)	.820
Body Mass Index (BMI)	0.70 (0.76)	.366	0.81 (0.78)	.306	0.70 (0.78)	.377	0.68 (0.6)	.265	0.68 (0.77)	.389	0.74 (0.70)	.298	1.00 (0.91)	.283	0.67 (0.61)	.283	1.08 (0.67)	.126
Disease onset (in months)			0.11 (0.13)	.380													−0.00 (0.1)	.968
STAI state					0.20 (0.44)	.657											0.18 (0.32)	.579
STAI trait					0.20 (0.44)	.664											−1.05 (0.38)	.014*
BDI‐II							1.19 (0.26)	<.001**									1.19 (0.4)	.009**
Sleep duration (h)									−1.28 (2.35)	.590							6.48 (2.93)	.042*
PSQI											1.61 (0.62)	0.014*					2.34 (0.88)	.017*
SF‐36 PF													0.01 (0.12)	.935			0.06 (0.09)	.539
SF‐36 RP													0.05 (0.09)	.629			0.08 (0.07)	.274
SF‐36 BP													−0.04 (0.21)	.863			−0.03 (0.15)	.861
SF‐36 GH													−0.08 (0.24)	.738			0.13 (0.19)	.497
SF‐36 VT													−0.43 (0.41)	.304			−0.51 (0.27)	.084
SF‐36 SF													0.05 (0.19)	.791			0.09 (0.14)	.525
SF‐36 RE													−0.06 (0.12)	.632			−0.16 (0.09)	.107
SF‐36 MH													−0.19 (0.38)	.623			0.22 (0.3)	.476
OHIP‐14															1.08 (0.25)	<0.001**	0.35 (0.30)	.265
*R* ^2^ (%)	.0	.858	.0	.852	.0	.868	31.2	.012*	.0	.894	5.7	.296	.0	.498	28.0	.020*	57.2	.009**
*R* ^2^ change (%)			.0	.381	.0	.522	31.2	<.001**	.0	.590	5.7	.014*	.0	.233	28.0	<.001**	57.2	.003**

*Note:* SE are the standard errors of the beta estimates. The *p*‐values were obtained from the hypothesis test on the regression coefficients.

Abbreviations: BDI, Beck's Depression Inventory; BMS, burning mouth syndrome; BP, bodily pain; BPI, brief pain inventory; ESS, Epworth Sleepiness Scale; GH, general health; MH, mental health; OHIP‐14, Oral Health Impact Profile‐14; PD‐Q, PainDETECT Questionnaire; PF, physical function; PSQI, Pittsburgh Sleep Quality Index; RE, role emotional; RP, role physical; SF, social functioning; SF‐36, 36 items Short‐Form Survey; SF‐MPQ, Short‐form McGill Pain Questionnaire; STAI, State–Trait Anxiety Inventory; VAS, visual analogue scale; VT, vitality.

*Moderately significant .01 < *p*‐Value ≤ .05. **Strongly significant *p*‐Value ≤ .01.

The final full model (model 9) tests the contribution of all the demographic variables and confounding factors, entered simultaneously, to pain. In SF‐MPQ, in BPI severity and in BPI interference the final model was found to be statistically significant (DR2 = 39.4% *p*‐value: .045*; DR2 = 52.1% *p*‐value: .011*; and DR2 = 57.2% *p*‐value: .003**, respectively).

## 4 DISCUSSION

Pain, psychiatric comorbidity and sleep disturbance are frequently overlapping and intertwined in BMS, resulting in a complex symptomatology.^8^, ^17^, ^33^ Therefore, the decision to carry out a detailed assessment, including the evaluation of all these features through appropriate questionnaires, is essential for BMS diagnosis and treatment, as suggested by the recent IMMPACT recommendations.^34^ The present study provides a wide evaluation of the pain experience and psychological profile in a sample of patients with BMS analysing the intensity, quality, severity and interference of pain on daily life activities and evaluating all the predictors that contribute to the pain experience.

In this study, pain was described by all the patients as burning in quality and it was considered the worst symptom by 23 (57%). The most frequent additional symptoms reported by our samples were xerostomia, a change in the tongue morphology and dysgeusia, in line with previous studies.^3^, ^33^, ^35^ In addition, the BMS patients suffered from a high pain intensity (VAS: 8–10; BPI intensity: 19.8–30), which deeply interfered, in terms of intensity and quality, in the individual's life activities (BPI interference: 9–32). Specifically, the scores of the VAS and BPI intensity of the BMS patients in our sample were in line with the study of Rezazadeh et al.^17^ but higher compared with other previous studies. Indeed, in the study of Braud et al.,^16^ which considered 17 BMS patients, the VAS median score ranged from 6 to 8, while in the study of Lee et al.^15^ on 65 patients the mean of the VAS intensity and BPI intensity was 5.0 (SD 2.4) and 4.2 (SD 2.6), respectively.

Instead, in this study, from the analysis of the pain quality, the median and IQR of the SF‐MPQ assessment was 5 [3–7.25] in the BMS patients, a result considered to be elevated, as compared with the study of Riordain^8^ on 32 individuals, who found a median and IQR for the SF‐MPQ of 1.0 [0.0–2.0], and with the study of Tu et al.^36^ on 248 patients, where the mean of the SF‐MPQ was 2.58 ± 2.98. Regarding the interference of pain, the median and IQR of the BPI interference score in our sample was higher (18 [9–32] compared with the recent study of Lee et al.^15^ (4.4; SD 3.1).^15^ The results of this study could be attributed to the delay in the diagnosis and, subsequently, to the persistence of not‐treated long‐lasting pain. Indeed, in line with the study of Lee et al.,^37^ in which the mean (SD) pain duration was 21.8 (30.7) months, and the study of Mignogna et al.,^38^ in which the average delay from the onset of the symptoms to a definitive diagnosis was 34 months, our study also confirms that BMS patients undergo a delay in diagnosis with an average of 2 years, confirming the poor knowledge about this disease among the medical community.

Moreover, the median and IQR of the PD‐Q score of the BMS patients in this sample was 8 [4.75–11.2] and only in 3 patients (7.5%) could the pain be considered as neuropathic (PD‐Q > 19). This result is in accordance with the study of Boku et al.^39^ on 29 patients with BMS where the authors concluded that the pain in fewer than 15% could include neuropathic pain elements. These results are in contrast with the study of Lopez‐Jornet et al.,^18^ on 33 BMS patients, where the pain was considered neuropathic in 21% of patients. In the present study, the analysis of the psychological profiles showed statistically significant differences in the STAI‐T, STAI‐S and BDI‐II scores between the BMS patients and the healthy subjects. In detail, 37 patients (92.5%) and 34 patients (85%) with BMS presented state and trait anxiety, respectively; the majority of the BMS patients (19; 45%) suffered from mild depression while 10 patients (25%) and 3 patients (7.5%) presented moderate and severe depression, respectively.

These results suggested a high prevalence of anxiety and depression in BMS patients in accordance with a recent systematic review and recent observational studies.^40^, ^41^, ^42^ In addition, the results of the present study demonstrated a strong positive correlation between anxiety and depression with pain in the bivariate analysis (*p*‐value < .001**). However, in the multiple regression analysis this correlation of both mood disorders remained significant only when considering the quality of pain (SF‐MPQ); at the same time, depression strongly correlated with PD‐Q, BPI severity and interference. In accordance with the study of Schiavone et al.,^42^ these results suggested that a high level of depression rather than anxiety can greatly affect the intensity, quality and interference of pain, confirming that depression is the most important predictor of the quality and interference of pain, as suggested by the stepwise selection in the regression analysis.

Regarding sleep evaluation, in line with the studies of Adamo et al.,^20^ the majority of the BMS patients in this study were poor sleepers (30; 75%) while the ESS scores did not reveal any differences between the cases and controls. In addition, the PSQI strongly correlated with the intensity (VAS, BPI severity) and quality of pain (SF‐MPQ) in the bivariate analysis. However, in the multiple regression it remained significant only in relation to the BPI severity.

Anxiety, depression and sleep disturbance are the most important critical psychological factors, which contribute to the modulation and aggravation of the pain experience in BMS, as suggested in several previous studies. Although mood disorders, poor sleep and pain may individually increase the other conditions, all these factors combined lead to a vicious cycle that perpetuates all the other problems.^15^, ^43^ The causal relationships among these comorbidities in BMS remain unclear, in the sense that we do not know which of these conditions begins first. Indeed, the final step, which considers the three most significant models of pain prediction (SF‐MPQ and BPI severity and interference), when all the variables are simultaneously included, could explain only 39.4%, 52.1% and 57.25% of the variance of the quality, severity and interference of the pain, respectively. This finding suggests that, although there is a high frequency of mood disorders in BMS patients and mood and pain share biological pathways, the processing of pain and mood may be distinct and, therefore, these conditions should be evaluated separately to better understand every feature of a patient affected by BMS.

Moreover, in line with a recent systematic review of Pereira et al.,^44^ a poor HRQoL and OHQoL in BMS patients has been detected in this study. Specifically, although in the bivariate analysis every component of the SF‐36 and OHIP‐14 strongly correlated with all the scales of pain, in a stepwise selection of the regression analysis only the OHIP‐14 remained strongly significant for the SF‐MPQ, PD‐Q and BPI interference. Therefore, in line with previous studies,^45^, ^46^ these results confirm a direct correlation between pain and OHQoL, taking into account the fact that interchanging symptoms and functional health perception can influence a low OHRQoL and increase xerostomia, dysgeusia and other local sensitivities. These results suggested that a tool assessing OHQoL, such as OHIP‐14 is essential, and it should be always included in the evaluation of patients with BMS in order to better understand the bidirectional correlation between pain and self‐perceived oral health. Indeed, poor OHQoL may affect social function of individuals causing in turn a psychological impairment and pain worsening.^46^

Pain dimensions continue to be one of the most frequently assessed outcome domains in every study about BMS.^9^, ^18^, ^36^, ^39^, ^42^ Therefore, a comprehensive evaluation using not only unidimensional scales, such as the VAS, should be an essential prerequisite in future studies on this syndrome. Indeed, the VAS scale, on account of its simplicity of use, gives a quick pain intensity analysis but the assessment should be completed with the use of multidimensional scales such as the SF‐MPQ and BPI, useful to evaluate the pain characteristics and the interference caused by the pain.^23^ In addition, these scales have shown their positive correlation with the psychological profile and QoL. Instead, in accordance with the study of Boku et al.,^39^ the PD‐Q seems not to be a suitable tool for the evaluation of pain in BMS, if different tools are available, because our results confirm that the nature of pain may be nociceptive rather than neuropathic.

The BPI is a simple tool, effective in the measurement of pain interference, intensity, location and, possibly, the effectiveness of the therapy.^14^ This questionnaire showed a good reliability and validity in several studies on chronic pain and could be useful in the pain assessment of patients with BMS because it gives information about how the psychosocial profile and pain affect and interfere with the individual's daily functioning, also influencing consequently his/her QoL.

Moreover, considering that QoL was the second most important domain according to the IMPACCT recommendations, clinicians should consider the SF‐36 and OHIP‐14 as additional tools to complete the analysis.^47^ It is difficult to select a unique questionnaire among these used because, despite the fact that in this study the OHIP‐14 was the tool more closely correlated with several components of the pain, it is also appropriate to consider the SF‐36 as a more complete questionnaire, effective in evaluating every component of the QoL.

Regarding the evaluation of the psychological profile, the choice of using self‐administration tools, such as the BDI‐II, STAI, PSQI and ESS, seems to be adequate in the assessment of anxiety, depression and sleep disturbance in BMS.^7^ Indeed, these questionnaires are simple to use and have demonstrated a good correlation with pain. In particular, as suggested by our results, it must be emphasised that depression was the most important predictor of pain and, therefore, it should be taken into account that a high level of depression may increase the perception of the intensity of the pain and its interference in the individual's daily life, worsening the QoL of BMS patients. In turn, the modulation of pain may improve the psychological well‐being, health function and QoL in patients with BMS.

The study presents some limitations. First, the small sample of the study, consisting predominantly of women and performed in a single centre, should be acknowledged. The limited sample size of this study does not allow the obtainment of a complete pain assessment and an understanding of the relative contribution of each feature. Secondly, the pain scales used were not able to evaluate the pain during the last 6 months and the differences in the duration of the disease may have influenced our results. Thirdly, it was not clear whether the onset of the sleep disturbance and mood disorders was antecedent to the pain, or the contrary, and no pain catastrophising scales were used. Moreover, the study started previously of RDC criteria publication^48^ and it included only SF‐MPQ as suggested tool. The effectiveness of this research is, therefore, exploratory and should be interpreted with care on account of the small size of the sample. Future studies with larger samples of BMS patients are needed.

## 5 CONCLUSIONS

The results of the current study show that patients with BMS suffer a high level of pain and that this type of pain, in terms of intensity and quality, seriously interferes with the individual's life activities. Mood disorders, sleep quality and QoL are differently correlated with the pain questionnaires and these factors may contribute to the pain experience. However, the significance of the role of the psychological constructs on the pain experience remains unclear.

In particular, depression could play a critical role in the pain experience of BMS patients. Conversely, demographic and risk factors, BMI, disease onset and sleep duration did not correlate with pain.

The complexity of the symptomatology in BMS patients dictates the need to evaluate the intensity of pain using not only unidimensional tools, such as the VAS, but also multidimensional questionnaires, such as the SF‐MPQ and BPI, in order to better understand the quality and interference of pain. In addition, self‐administration questionnaires, such as the BDI‐II, STAI, PSQI, ESS, SF‐36 and OHIP‐14 should be considered for a comprehensive assessment of the psychological profile and QoL in patients affected by BMS.

## AUTHORS CONTRIBUTIONS

FC and DA conceptualised the study, EC defined the methodology; FC and EC performed the data collection; LDA and MA performed the statistical analysis; GP, GM and PS participated in conceptualisation of the study and participated in manuscript editing. FC, EC and DA drafted the manuscript; MDM, DA and GP participated in defining the methodology and in manuscript editing; MDM and DA contributing to the scope of discussion. All authors approved the final version of the manuscript.

## CONFLICT OF INTEREST

The authors have no conflict of interest, no financial support and no off‐label or investigational use to declare.

## Supporting information


Figure S1

## Data Availability

Data are available on request from the authors—The data that support the findings of this study are available from the corresponding author upon reasonable request.
