# Supplementary material for: The use of self‐report questionnaires in an analysis of the multidimensional aspects of pain and a correlation with the psychological profile and quality of life in patients with burning mouth syndrome: A case‐control study
Source: J Oral Rehabil. 2022 Jun 21;49(9):890–914. doi: 10.1111/joor.13343 (PMC9544557; doi:10.1111/joor.13343)
Supplement: Supplementary file 1 — Figure S1 [file JOOR-49-890-s001.pptx]

## Slide 1
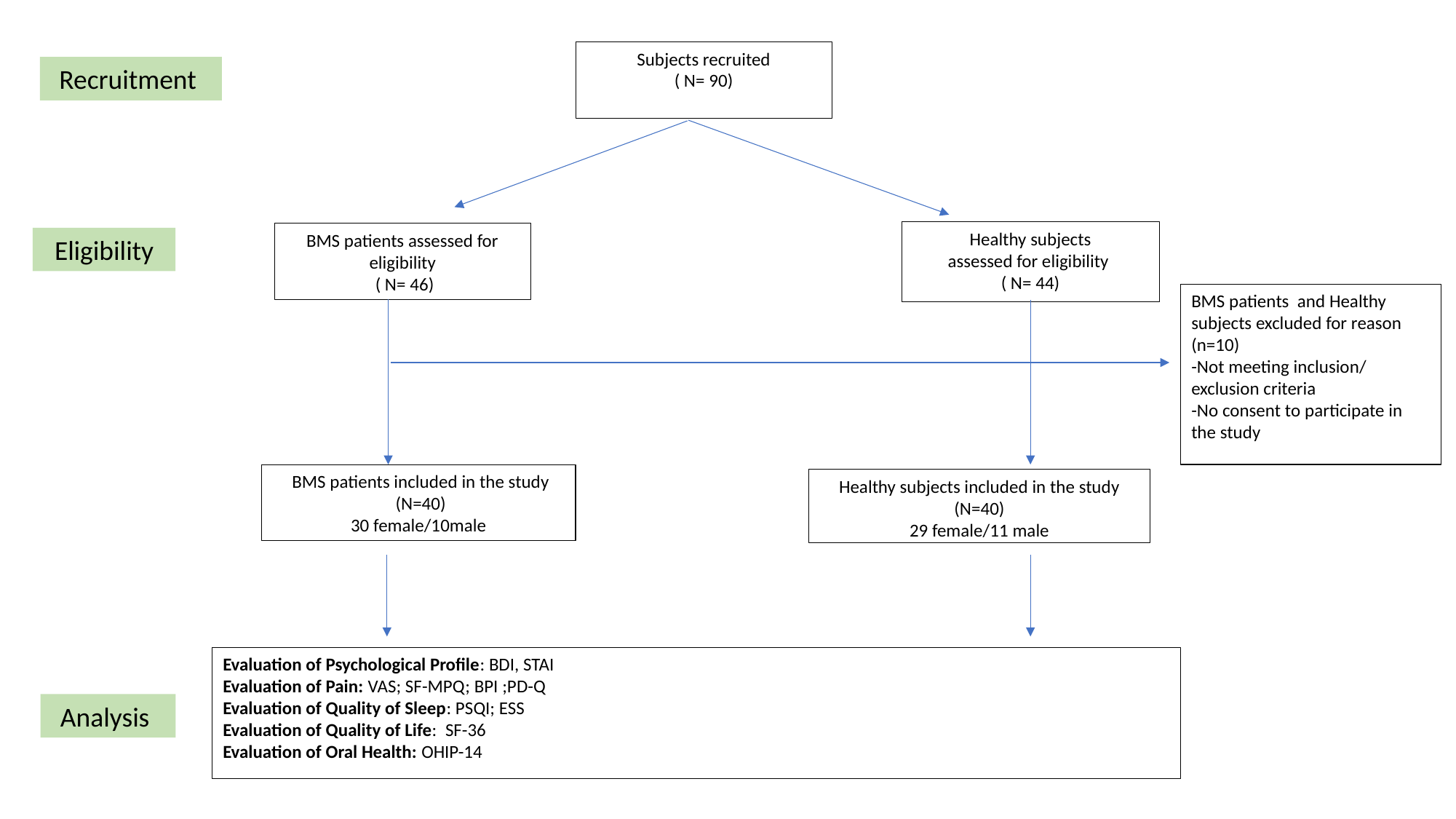

Subjects recruited
( N= 90)
Recruitment
Healthy subjects
assessed for eligibility
( N= 44)
BMS patients assessed for eligibility
 ( N= 46)
Eligibility
BMS patients and Healthy subjects excluded for reason (n=10)
-Not meeting inclusion/ exclusion criteria
-No consent to participate in the study
 BMS patients included in the study
 (N=40)
30 female/10male
 Healthy subjects included in the study
(N=40)
29 female/11 male
Evaluation of Psychological Profile: BDI, STAI
Evaluation of Pain: VAS; SF-MPQ; BPI ;PD-Q
Evaluation of Quality of Sleep: PSQI; ESS
Evaluation of Quality of Life: SF-36
Evaluation of Oral Health: OHIP-14
Analysis
